# Chromophobe renal cell carcinoma with concomitant sarcomatoid transformation and osseous metaplasia: a case report

**DOI:** 10.1186/1471-2490-13-72

**Published:** 2013-12-13

**Authors:** Yoshimi Tanaka, Takuya Koie, Shingo Hatakeyama, Yasuhiro Hashimoto, Chikara Ohyama

**Affiliations:** 1 Department of Urology, Hirosaki University Graduate School of Medicine, 5 Zaifucho, Hirosaki 036-8562, Japan

**Keywords:** Chromophobe renal cell carcinoma, Sarcomatoid change, Osseous metaplasia

## Abstract

**Background:**

Chromophobe renal cell carcinoma is the third most common form of adult renal epithelial neoplasm. A sarcomatoid component occurs in approximately 8% of all chromophobe renal cell carcinoma cases, while metaplastic bone formation is extremely rare.

**Case presentation:**

An abdominal computed tomography scan revealed a hypovascular tumor with focal calcification, measuring 2.5 × 2.3 cm, in the upper pole of the right kidney. The tumor was clinically diagnosed as a right renal cell carcinoma that showed signs of calcification, and a laparoscopic right radical nephrectomy was performed. The cut surface of the tumor was beige in color and indicated that the tumor was an extensively ossified mass. Histological analysis revealed three distinct morphological components of the tumor. The chromophobe renal cell carcinoma consisted of compact epithelial cells arranged in a nested pattern, and these were mixed with extensive areas of sarcomatoid spindle cells with marked nuclear pleomorphism and brisk mitotic activity. The tumor also contained multiple foci of metaplastic ossification.

**Conclusion:**

Chromophobe renal cell carcinoma with concomitant osseous metaplasia and sarcomatoid transformation is a very rare occurrence.

## Background

Chromophobe renal cell carcinoma (CRCC) is the third most common form of adult renal epithelial neoplasm, accounting for 5.9% of all RCC cases [1], and has a better prognosis than clear cell carcinoma. The 5- and 10-year cancer-specific survival rates have been reported to be 100% and 90%, respectively [1], although sarcomatoid differentiation of CRCC is generally associated with a worse prognosis [2,3]. Malignant tumors with sarcomatoid change had a 35% and 27%, 5-year disease-specific and progression-free survival, respectively [1]. The RCC exhibits various associated secondary changes that include necrosis, hemorrhage, edema, fibrosis, and calcification. A sarcomatoid component occurs in approximately 8% of all CRCC cases [2], while metaplastic bone formation is extremely rare [4]. Here, we report a unique case of CRCC with concomitant sarcomatoid transformation and massive osseous metaplasia.

## Case presentation

**Figure 1:**
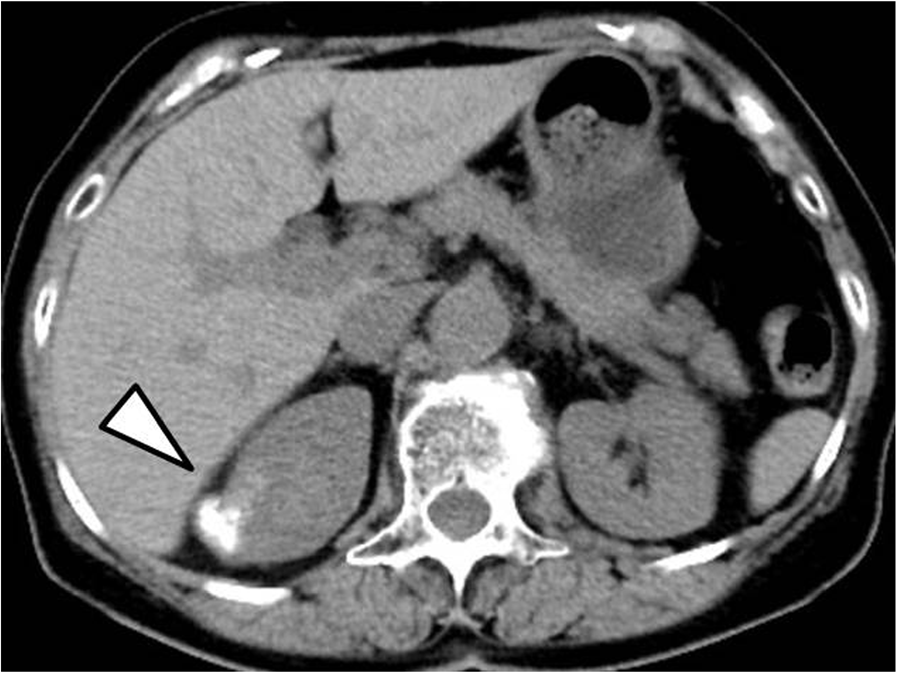
**Plain abdominal computed tomography (CT).** Abdominal CT revealed a hypovascular tumor with massive calcification, measuring 2.5 × 2.3 cm, in the upper pole of the right kidney (arrow).

**Figure 2:**
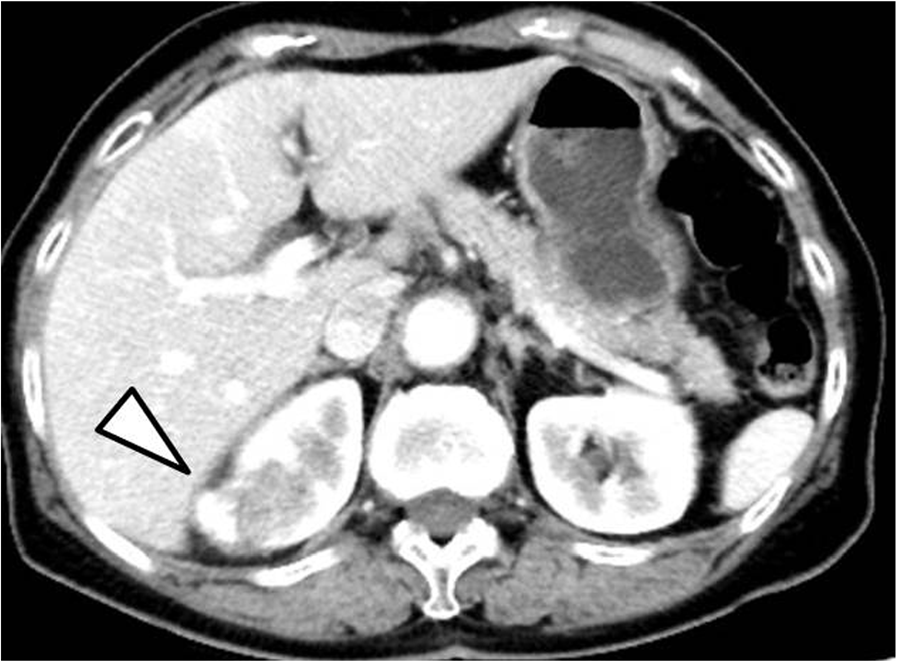
**Enhanced abdominal computed tomography (CT).** Abdominal CT revealed a hypovascular tumor with massive calcification, measuring 2.5 × 2.3 cm, in the upper pole of the right kidney (arrow).

**Figure 3:**
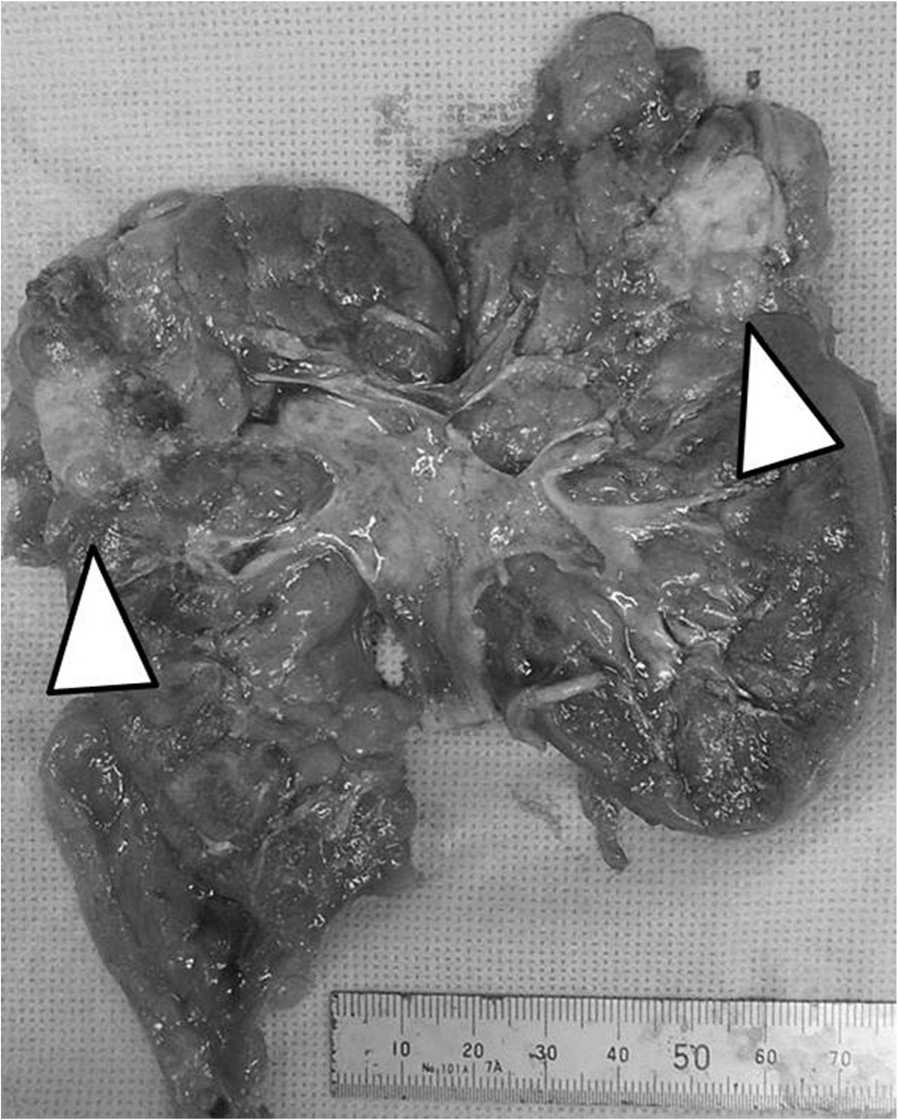
**Macroscopic findings.** Macroscopic examination revealed a solid, beige colored, and extensively ossified mass, measuring 3 × 2.5 cm, in the upper pole of the resected kidney (arrow).

A 77-year-old woman visited our hospital with an incidentally identified right renal tumor by a screening abdominal ultrasonography procedure for hepatitis C infection. All laboratory test results were within normal limits. An abdominal computed tomography revealed a hypovascular tumor with focal calcification, measuring 2.5 × 2.3 cm, in the upper pole of the right kidney (Figures 1 and 2). The tumor was clinically diagnosed as a right RCC with calcification and was classified as cT1aN0M0, according to the tumor-node-metastasis system [5]. The patients underwent the implantation of a pacemaker due to sick sinus syndrome and had poor ejection fraction. Laparoscopic right radical nephrectomy was performed. The cut surface of the tumor was beige in color and indicated that the tumor was an extensively ossified mass (Figure 3).

**Figure 4:**
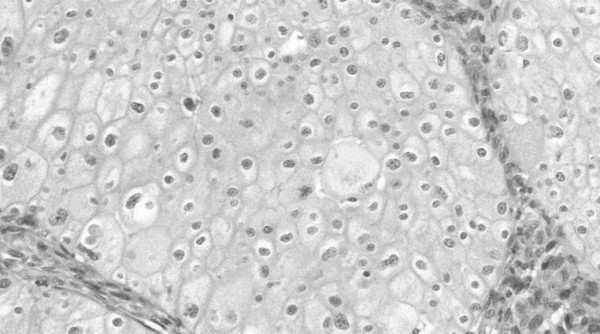
**Hematoxylin-eosin stained section.** (chromophobe renal cell carcinoma. The cells had eosinophilic cytoplasm with accentuated cell borders and centrally located nuclei that had wrinkled peripheral borders and varying degrees of hyperchromatism (magnification, ×20).

**Figure 5:**
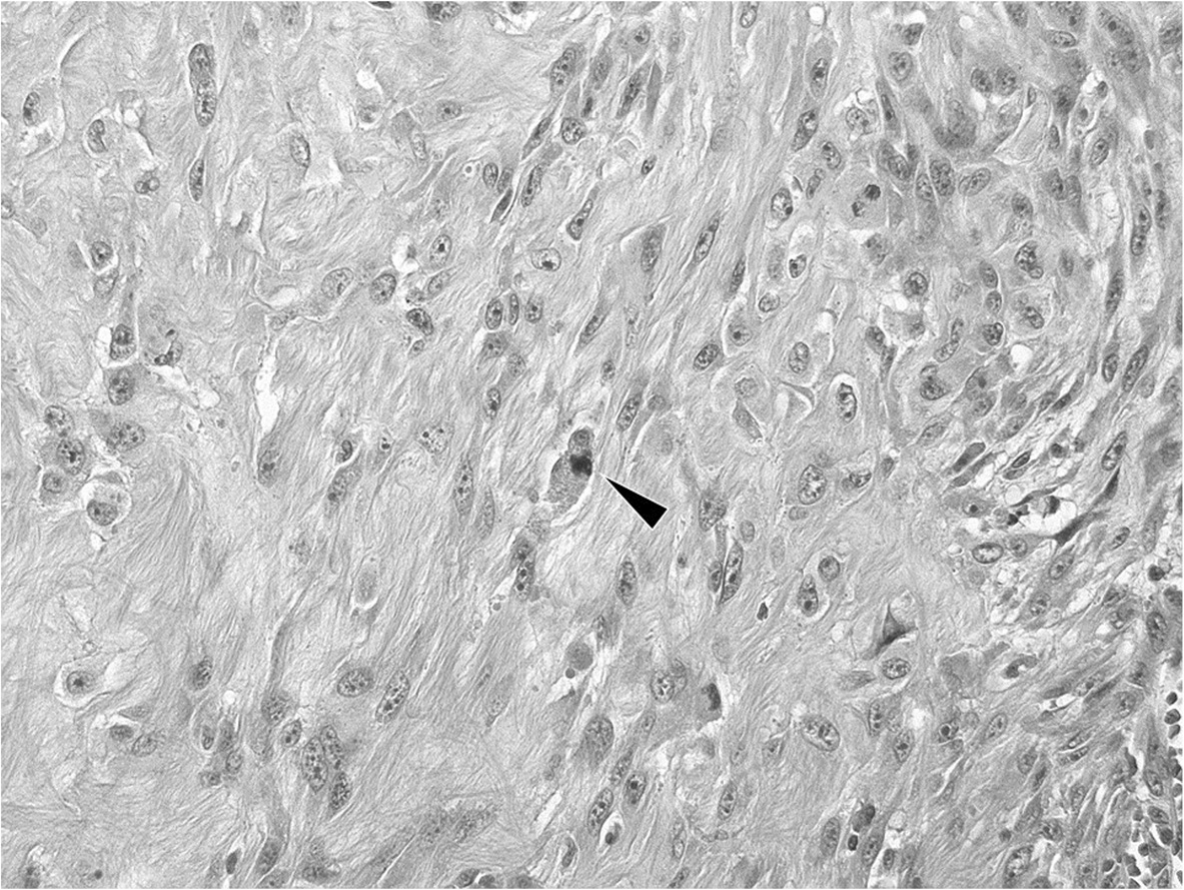
**Hematoxylin-eosin stained section.** (B: spindle cell component). The spindle cells were arranged in ill-defined fascicles that had a focal storiform pattern and mitosis (arrow) (magnification, ×200).

**Figure 6:**
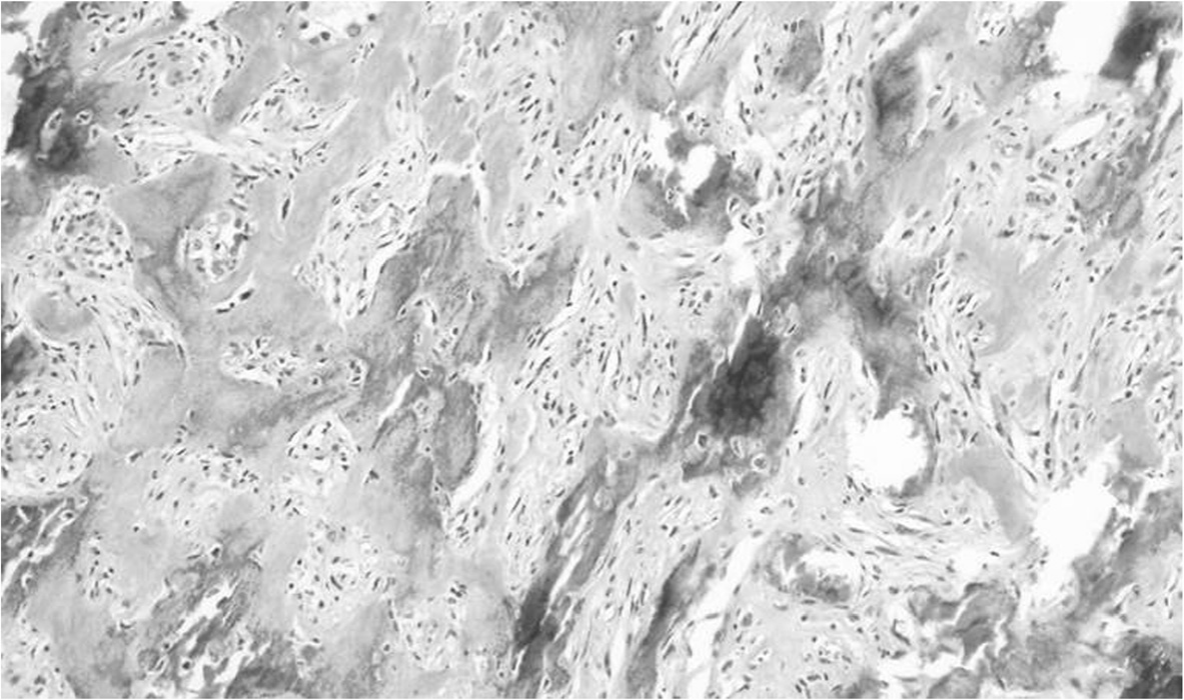
**Hematoxylin-eosin stained section.** (osseous metaplasia). Metaplastic ossification was apparent (magnification, ×20).

Histological analysis revealed three distinct morphologic components of the tumor, which consisted of compact epithelial cells arranged in a nested pattern (Figure 4). The cells contained eosinophilic cytoplasm with accentuated cell borders, and the centrally located nuclei had wrinkled peripheral borders and varying degrees of hyperchromatism. Admixed with the CRCC were extensive areas of sarcomatoid spindle cells with marked nuclear pleomorphism and brisk mitotic activity (Figure 5). The spindle cells were arranged in ill-defined fascicles with a focal storiform pattern, exhibiting an aggressive growth pattern with extracapsular invasion into the adipose tissue. Another feature of this tumor was the presence of multiple foci of metaplastic ossification (Figure 6). There were also hyaline degenerative change and fibrosis in all areas.

The patient is alive and free from disease 12 months after surgery.

### Discussion

To the best of our knowledge, this is the first reported case of CRCC with concomitant osseous metaplasia and sarcomatoid transformation.

A number of recent studies concerning the prognosis of patients with CRCC have reported relatively good survival rates for this disease [1,6], and cancer-related death occurred in 22%, 16%, and 8.6% of clear, papillary, and CRCC cases, respectively [7]. The good survival outcomes in these cases were probably because more than 70% of patients had organ-confined disease and more than 60% of tumors were low grade [7].

The incidence of sarcomatoid transformation is approximately 5% in RCC [8], increasing to 8% in the CRCC subtype [2,9]. Heterologous sarcomatoid components, such as chondrosarcomatous, osteosarcomatous or rhabdomyosarcomatous component have also been reported [10]. Akhtar et al. suggested that sarcomatoid development in CRCC might be associated with its peculiar genetic profile, which makes cells prone to hyperploidization [11]. Bruneli et al. demonstrated that both the epithelial and sarcomatoid elements in a CRCC have different genetic abnormalities and that the latter show multiple gains of chromosomes 1, 2, 6, 10, and 17 [12]. The coexistence of both CRCC and sarcomatoid carcinoma may be due to either the dedifferentiation of the more highly differentiated chromophobe cell tumor or the coincidental development of two synchronous tumors [13]. The former model is more widely accepted as it is based on the evolution of renal carcinoma into a spindle cell population. This sarcomatoid change may be the result of extensive chromosomal rearrangement, leading to identical spindle morphology [12].

Sarcomatoid CRCC is a more aggressive neoplasm and has a very poor prognosis compared with classic chromophobe carcinoma [4]. The presence of a sarcomatoid component has been reported to be associated with an increased risk of metastasis and an unfavorable prognosis [2,9,14]. Previously reported cases of CRCC with sarcomatoid differentiation have shown distant organ metastases to sites including the bone, liver, lung, and lymph nodes [14].

RCCs exhibit a number of changes such as hemorrhage, necrosis, fibrosis, and hyalinization. However, although calcification is a well-recognized feature of renal tumors, they are rarely ossified. Daniel reported that 10.3% of RCCs had calcified foci, based on a review of 2,709 renal masses at the Mayo Clinic [15]. Moreover, reports of histopathologically confirmed osseous metaplasia or bone formation within RCC are rare [16]. The presence of ossification in CRCC, however, was noted in four previously reported cases [4].

The mechanism of ossification is unclear, although it might involve a metaplastic or reparative response either in the tumor or in the surrounding tissue, the production of bone by tumor cells, or the ossification of a preexisting mucin or calcium deposit [4]. Osseous metaplasia may occur secondary to ischemia, necrosis, or inflammation in the tumor or surrounding tissues [4].

The prognostic significance of ossification in CRCC remains unclear because of the limited number of cases reported to date. However, several studies demonstrated that ossification is a significant prognostic marker for patients with RCC [4,17] and is usually representative of an early stage without invasion or metastasis [17]. Therefore, RCC with osseous metaplasia implies a more favorable prognosis, although this is contradicted by some reports, suggesting that ossification is actually associated with high-grade tumors and a poor prognosis [18].

## Conclusion

In conclusion, CRCC with concomitant osseous metaplasia and sarcomatoid transformation is a very rare occurrence. It is important to recognize that this unusual variant of renal cancer has the potential to behave aggressively and to metastasize.

### Consent

Written informed consent was obtained from the patient for publication of this case report and the accompanying images. A copy of the written consent is available for review by the Editor-in-Chief of this journal.

## Abbreviations

CRCC: Chromophobe renal cell carcinoma; RCC: Renal cell carcinoma.

## Competing interests

The authors declare that they have no competing interests.

## Authors’ contributions

YT drafted the manuscript. TK was involved in the drafting of the manuscript. SH performed the clinical follow-up and contributed to the manuscript. YH reviewed the pathological specimen. CO and TK performed the operation. CO was responsible for the conception and design of this study, interpretation of the data, and critical revision of the manuscript. All authors read and approved the final manuscript.

## Authors’ information

YT: Resident. TK: Associate professor. SH: Lecturer. YH: Associate professor. CO: Professor and Chairman. Department of Urology, Hirosaki University Graduate School of Medicine, Hirosaki, Japan.

## Pre-publication history

The pre-publication history for this paper can be accessed here:

http://www.biomedcentral.com/1471-2490/13/72/prepub
